# On the Convergence of Internet of Things and Decentralized Finance: Security Challenges and Future Directions

**DOI:** 10.3390/s26061740

**Published:** 2026-03-10

**Authors:** Prasannakumaran Sarasijanayanan, Nithya Nedungadi, Sriram Sankaran

**Affiliations:** Center for Cybersecurity Systems and Networks, Amrita Vishwa Vidyapeetham, Amritapuri 690525, India

**Keywords:** Internet of Things (IoT), decentralized finance (DeFi), blockchain security, smart contracts, IoT security, oracles, decentralized applications (DApps)

## Abstract

The rapid convergence of the Internet of Things (IoT) and decentralized finance (DeFi) is reshaping the digital economy by enabling autonomous, trustless, and value-driven interactions among connected devices. This paper provides a comprehensive survey of the emerging paradigm that combines IoT’s pervasive sensing and communication capabilities with DeFi’s programmable financial infrastructure. We first discuss the motivation behind this convergence and explore key opportunities, including autonomous machine-to-machine (M2M) payments, decentralized data marketplaces, and trustless IoT service provisioning. Despite its potential, IoT–DeFi integration introduces significant security and privacy challenges related to smart contract vulnerabilities, consensus protocol risks, oracle manipulation, and constrained device capabilities. We review existing mitigation approaches such as lightweight cryptography, secure contract design, and decentralized identity management, and critically assess their limitations in heterogeneous, resource-limited environments. Building on this analysis, identify research gaps and propose future directions emphasizing formal verification of IoT-integrated smart contracts, robust oracle design, interoperability frameworks, and privacy-preserving trust models. This survey systematically maps opportunities, threats, and open issues. In doing so, it guides researchers and practitioners toward building secure, scalable, and energy-efficient IoT–DeFi ecosystems for next-generation decentralized applications.

## 1. Introduction

The rapid proliferation of the Internet of Things (IoT) and the rise of decentralized finance (DeFi) represent two parallel technological developments that are increasingly intersecting. IoT systems deploy billions of connected devices that continuously sense, act, and exchange data across diverse environments such as industrial automation, supply chains, smart mobility, and healthcare. DeFi, by contrast, provides programmable and non-custodial financial infrastructure through blockchain smart contracts. These smart contracts enable a wide range of financial services, including decentralized lending, trading, micropayments, and automated asset management. The convergence of these domains enables new machine-to-machine (M2M) economic interactions, yet it also introduces a complex set of security, interoperability, and economic challenges that remain inadequately understood.

While prior work has surveyed IoT security issues and DeFi vulnerabilities independently, the literature still lacks a comprehensive examination of the combined attack surface and operational risks. These challenges arise when resource-constrained IoT devices interact with immutable, composable, and financially exposed DeFi protocols. Existing surveys typically discuss blockchain-based IoT applications or DeFi security in isolation, but do not articulate (i) how IoT constraints challenge financial smart contract execution, (ii) how DeFi composability amplifies risks introduced by unreliable or manipulable IoT data, or (iii) what system-level design principles are needed to securely integrate the two ecosystems. This gap motivates a focused survey that not only synthesizes existing work but also identifies unresolved theoretical and practical issues at the IoT and DeFi intersection.

### 1.1. Problem Statement and Research Questions

Opportunities such as autonomous M2M payments, decentralized data marketplaces, and incentive-aligned device coordination make IoT–DeFi convergence highly attractive. Yet, this integration remains challenging due to security risks that stem from both IoT and DeFi ecosystems. IoT devices often lack sufficient computational, memory, and energy resources to support strong cryptographic operations or to maintain secure firmware lifecycles. DeFi protocols, meanwhile, are vulnerable to smart contract exploits, oracle manipulation, composability-induced attack propagation, and governance-based adversarial control. When coupled together, these weaknesses compound the following: compromised devices can trigger financial losses, and adversarial economic incentives can motivate attacks on physical IoT infrastructure.

To address these concerns, this survey investigates the following research questions (RQs):

- RQ1: What are the dominant attack surfaces and systemic risks that emerge when IoT infrastructures interact with DeFi protocols? The above analysis demonstrates how systemic risks arise from cascading failures across IoT, oracle, and DeFi layers.
- RQ2: How do resource constraints, device heterogeneity, and data provenance challenges in IoT affect the security and correctness of DeFi smart contracts and oracles?
- RQ3: What mitigation strategies, including cryptographic, architectural, protocol-level, or governance-based approaches, are most effective for securing IoT and DeFi integration? This evaluation identifies mitigation strategies that are most effective under realistic IoT–DeFi deployment constraints.
- RQ4: What open research gaps must be addressed to design scalable, trustworthy, and economically viable IoT and DeFi systems?

These questions define the scope of this survey and guide its structure.

### 1.2. Scope and Objectives

The scope of this survey is limited to architectures, protocols, and security mechanisms that enable direct or mediated interaction between IoT devices and DeFi systems. This includes smart contracts, on-chain oracles, blockchain-based markets, and tokenized data flows. Our scope omits broader blockchain–IoT integrations unrelated to financial mechanisms and excludes DeFi use cases that do not depend on data originating from the physical world. Instead, the objectives of this survey are as follows:

- To characterize the security challenges unique to IoT and DeFi convergence, beyond traditional IoT or DeFi-only threat models;
- To analyze how device constraints, oracle reliability, smart contract attack vectors, and financial contagion interact across layers;
- To review and systematize existing mitigation mechanisms spanning lightweight cryptography, trusted hardware, secure smart contract design, and decentralized identity frameworks;
- To identify theoretical gaps, including data trustworthiness models, adaptive reputation systems, economic incentive misalignment, and formal verification for IoT-integrated contracts;
- To outline forward-looking research directions that can support secure, scalable, and economically viable IoT and DeFi ecosystems.

### 1.3. Contributions and Novelty of This Survey

This survey makes the following contributions:

- First security-focused synthesis of IoT and DeFi convergence: Unlike prior surveys that examine IoT and blockchain or DeFi and security separately, this work explicitly analyzes the compounded attack surface that arises when IoT devices interact with composable financial contracts.
- A unified taxonomy of IoT and DeFi integration models: We propose a novel taxonomy covering architectural patterns, data-flow models, incentive mechanisms, oracle dependencies, and supported IoT verticals.
- Cross-domain threat analysis: We map IoT vulnerabilities, including device capture, firmware compromise, and physical tampering, to their potential financial impact in DeFi systems, including cascading effects through composability.
- Comprehensive review of mitigation strategies: We evaluate lightweight cryptography, formal verification, federated learning, decentralized identity, trusted hardware, and Layer-2 scalability with respect to IoT feasibility.
- Identification of empirical and theoretical research gaps: We highlight deficiencies in current oracle designs, identity frameworks, interoperability models, and secure integration testing methodologies.

Overall, this survey goes beyond general background descriptions and provides a structured, gap-driven, and security-centric analysis of IoT and DeFi convergence. Table 1 presents the proposed IoT–DeFi taxonomy, outlining the key dimensions used to classify integration approaches and use cases. The taxonomy provides a structured framework covering architecture, data flow models, incentive mechanisms, consensus protocols, security features, and supported IoT verticals, facilitating systematic comparison and identification of research gaps [1].

### 1.4. Organization of the Paper

This survey provides a comprehensive overview of the convergence between the Internet of Things (IoT) and decentralized finance (DeFi), two rapidly evolving domains with the potential to reshape digital ecosystems. The paper begins by introducing the core concepts and motivations for integrating IoT and DeFi. Then it provides a detailed background on their technological foundations, including blockchain, smart contracts, and IoT communication models. A novel framework is proposed to classify IoT–DeFi integration approaches based on architectural patterns, device roles, and interaction models. Real-world and theoretical applications such as machine-to-machine (M2M) payments, decentralized data marketplaces, and autonomous energy trading are explored to highlight the practical potential of this convergence. The survey then reviews existing system architectures and integration frameworks, analyzing their strengths and limitations. Critical security and privacy challenges are discussed, including vulnerabilities in smart contracts and IoT endpoints, along with potential mitigation strategies. In addition, the paper identifies key technical challenges such as scalability, resource constraints, and regulatory concerns. Finally, it outlines current research trends and suggests future directions, emphasizing the need for lightweight protocols, cross-domain standardization, and secure data sharing mechanisms to realize the full potential of IoT–DeFi systems.

## 2. Background and Related Work

### 2.1. Methodological Approach to the Literature Review

To ensure a structured and reproducible analysis of the research landscape, this section adopts a systematic literature review (SLR) methodology. We queried ACM Digital Library, IEEE Xplore, Scopus, Web of Science, and arXiv between 2015 and 2025, using combinations of the following keywords: “IoT security”, “blockchain IoT”, “DeFi security”, “IoT DeFi integration”, “machine-to-machine payments”, “oracle security”, “blockchain-based IoT” and “tokenized data”.

Inclusion criteria included (i) peer-reviewed or preprint works addressing IoT–blockchain or DeFi security, (ii) architectures or protocols enabling IoT–DeFi interactions, (iii) surveys on IoT, blockchain, or DeFi technologies, and (iv) foundational works on smart contracts, oracles, identity, or distributed consensus. Exclusion criteria excluded papers focusing only on cryptocurrency price analysis, blockchain governance unrelated to IoT or DeFi, and IoT applications with no cryptographic or economic components.

After initial retrieval of 842 papers and removal of duplicates, 228 papers remained for title and abstract screening, and 112 were assessed in full. From these, 87 papers were included in our final synthesis. The review follows an analytical framework with three dimensions, as follows: (i) architectural integration models, (ii) security assumptions and threat models, and (iii) economic and operational constraints. This framework allows comparison across heterogeneous literature rather than descriptive summarization.

### 2.2. Overview of the Internet of Things (IoT)

IoT refers to a distributed ecosystem of interconnected devices equipped with sensors, actuators, and lightweight communication modules. These devices operate across layered architectures comprising edge nodes, gateways, and cloud backends [2]. Foundational IoT research [3] established the conceptual models for device-to-cloud communication, hierarchical processing pipelines, and the importance of scalability and interoperability.

However, despite its ubiquity, IoT faces persistent issues related to centralized data ownership, siloed architectures, weak device identity models, and limited cryptographic capacity. Recent studies (e.g., [4]) highlight systemic vulnerabilities due to outdated firmware, poor authentication, and a lack of secure data provenance. These constraints become acute when IoT systems interact with financial infrastructures that require verifiable, high-integrity data streams.

### 2.3. Overview of Decentralized Finance (DeFi)

DeFi leverages blockchain infrastructures to automate financial services through smart contracts [5]. Foundational platforms such as Ethereum established programmability via the EVM, enabling lending protocols (Aave), automated market makers (Uniswap), and decentralized stablecoins (DAI/MakerDAO). Research such as [6,7] categorizes threats, including oracle manipulation, flash-loan attacks, composability risks, and governance-based takeovers. Despite rapid growth, DeFi remains vulnerable to small code defects and flawed assumptions about data integrity, making it risky to integrate with IoT systems that may supply noisy or adversarial sensor data.

### 2.4. Motivation for IoT–DeFi Integration

**Figure 1:**
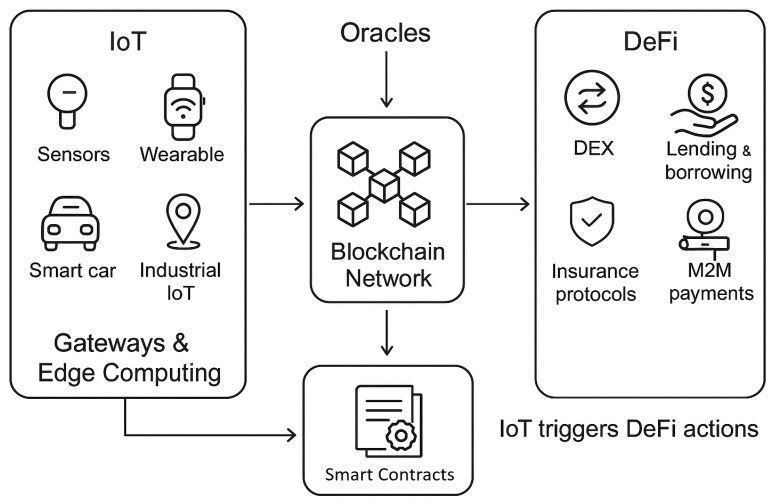
Conceptual integration of IoT and DeFi via blockchain, oracles, and smart contracts.

Integrating IoT with DeFi enables trustless automation, micropayments, data monetization, decentralized energy markets, and financialized machine-to-machine interactions [8,9,10]. Prior literature highlights potential synergies, but often lacks a security-centric or economically grounded analysis. Existing research inconsistently addresses foundational requirements for such integration, including secure oracles, provenance-aware data flows, incentive-compatible microtransactions, and resource-aware smart contract execution. Figure 1 illustrates the complete integration workflow involving IoT sensors that collect real-time data, gateway devices that transmit this data to the blockchain, oracle services that bridge off-chain and on-chain information, blockchain networks that ensure secure and immutable processing, smart-contract-based DeFi platforms that automate financial logic, and M2M payment mechanisms that enable autonomous machine-to-machine transactions.

### 2.5. Thematic Synthesis of Prior Work

#### 2.5.1. Blockchain as a Trust Layer for IoT

Studies such as [11] explore blockchain for device identity, auditability, and decentralized trust. These works propose lightweight consensus algorithms, DAG-based structures [12], and incentive-driven networks (e.g., Helium [13]). Yet, most do not consider the financial exposure or attack amplification when IoT data triggers DeFi transactions.

#### 2.5.2. Smart Contract Security and DeFi Risk Models

Surveys [4] analyze smart contract vulnerabilities, highlighting issues such as reentrancy, integer overflows, access-control flaws, and logic inconsistencies. Complementary studies [14] focus on formal verification techniques, automated vulnerability detection, and the construction of benchmark datasets used to evaluate the robustness of contract analysis tools. DeFi-oriented reviews (e.g., [15]) further examine auditing frameworks, runtime monitoring approaches, and forensic analyses of past attacks to characterize protocol-level risks such as MEV, oracle manipulation, or liquidity exploits. However, these bodies of work primarily assess smart contracts and DeFi risks in isolation from external data ecosystems. They rarely consider how IoT-specific factors—such as noisy sensor measurements, intermittent connectivity, temporal drift, device heterogeneity, or intentional data tampering—affect the correctness of IoT data used to trigger on-chain financial actions.

#### 2.5.3. IoT–Blockchain Data Marketplaces and Tokenized Data

Frameworks such as the Ocean Protocol [16] and federated learning models [17] explore decentralized data monetization by enabling IoT-generated information to be shared, exchanged, and priced in open marketplaces. These platforms introduce economic incentives for data contributors and establish mechanisms for secure access control, provenance tracking, and privacy preservation. However, existing approaches primarily focus on operational aspects of data sharing rather than the broader financial implications. In particular, they do not investigate the systemic risks that emerge when tokenized IoT data transitions from a mere digital commodity to a financial asset actively integrated into DeFi protocols. Such integration raises new concerns related to data-driven market manipulation, valuation instability, oracles’ attack surfaces, and cascading failures when corrupted or low-quality physical-world data propagates into automated financial systems.

#### 2.5.4. Early Architectures for IoT–Blockchain Payments

Foundational studies such as Dorri et al. [18] and smart contract-based M2M payment systems [19] introduced micropayment protocols for IoT. Later work (e.g., [20]) demonstrated the real-world value of streaming sensor markets. Yet, these systems lack analysis of composable DeFi environments where adversarial sensor spoofing may propagate into financial contagion.

### 2.6. Critical Evaluation of Existing Surveys

Table 2 offers a detailed comparison of prior surveys. Most studies fall into one of three categories:

- IoT-focused surveys (e.g., [21]) emphasize device security, consensus mechanisms, and privacy, but do not examine financial incentives or DeFi risk propagation.
- DeFi-focused surveys (e.g., [7]) investigate attack vectors in financial protocols but ignore physical-world data dependencies.
- Blockchain–IoT surveys (e.g., [11]) discuss blockchain as middleware, but seldom analyze composability, economic models, or oracle vulnerabilities relevant to DeFi.

Critically, no existing survey offers a unified analysis of (i) cross-domain attack surfaces, (ii) IoT-induced financial risks, (iii) verifiable data flows feeding smart contracts, and (iv) economically sustainable IoT–DeFi integration. Our survey addresses this gap with a security-centric, economically aware, and architecture-level synthesis.

**Table 2:** Comparative summary of recent review papers related to IoT, blockchain, and DeFi security, including our work.

No.	Paper Reference	Year	Main Focus	IoT Coverage	DeFi Coverage	Key Contributions/Highlights
1	[22]	2023	Blockchain-based IoT security, privacy and trust frameworks	Strong	Limited	Explains blockchain as a trust layer for IoT; classifies attacks and mitigation by consensus mechanisms.
2	[23]	2023	DeFi security, smart contract vulnerability analysis	None	Strong	Reviews DeFi attack vectors and repair tools; focuses on security audits and automated vulnerability detection.
3	[15]	2023	Evaluation of DeFi security tools and their real-world applicability	None	Strong	Compares 20+ DeFi analysis tools; discusses gaps between research tools and developer needs.
4	[24]	2024	Smart contract vulnerabilities and detection methods	Indirect	Medium	Systematic mapping of vulnerabilities; survey detection, auditing, and verification frameworks.
5	[4]	2023	Blockchain + AI for IoT security	Strong	Limited	Integrates AI models with blockchain for IoT threat detection; identifies scalability and heterogeneity issues.
6	[25]	2025	Design patterns for secure and efficient smart contracts	None	Medium	Proposes secure design templates and anti-patterns to prevent known vulnerabilities in contracts.
7	[14]	2023	Smart contract vulnerability taxonomy and datasets	None	Medium	Reviews datasets, detection tools, and static/dynamic analysis methods for DeFi contract flaws.
8	[11]	2024	Blockchain-enabled IoT architectures and consensus mechanisms	Strong	Indirect	Evaluates blockchain–IoT consensus schemes; discusses latency, scalability and energy constraints.
9	[7]	2022	Comprehensive DeFi security overview	None	Strong	Classifies security threats, attack models, and financial risks in DeFi; suggests mitigation frameworks.
10	[26]	2024	AI-based blockchain–IoT security mechanisms	Strong	Indirect	Highlights AI-driven intrusion detection, trust computation, and anomaly detection in blockchain-IoT systems.
11	Our Work	2025	Secure and energy-efficient integration of IoT devices with DeFi protocols	Strong	Strong	Analyzes the convergence of IoT and DeFi, highlighting key opportunities, security challenges, and future research directions for secure and scalable integration.

## 3. Opportunities in the Convergence of IoT and DeFi

The convergence of the Internet of Things (IoT) and decentralized finance (DeFi) presents significant opportunities for creating autonomous, data-driven financial systems. IoT devices can seamlessly interact with DeFi platforms to enable real-time micropayments, automated billing, and decentralized data marketplaces. This integration enhances transparency, reduces the need for intermediaries, and allows for efficient, trustless transactions among machines and users. Applications span across sectors such as smart cities, supply chains, energy management, and healthcare, where IoT data can trigger smart contract-based financial operations, driving innovation and economic efficiency.

### 3.1. Autonomous Machine-to-Machine (M2M) Payments

Autonomous machine-to-machine (M2M) payments refer to the real-time exchange of value between devices without human intervention. Enabled by IoT, blockchain, and smart contracts, M2M payments allow machines—such as electric vehicles, smart appliances, and industrial robots—to autonomously negotiate and settle microtransactions for services like energy usage, data sharing, or maintenance. For instance, an electric vehicle can automatically pay a charging station in cryptocurrency after verifying energy consumption. Layer-2 solutions or off-chain aggregation are often required to ensure economic feasibility.

### 3.2. Decentralized Data Marketplaces

**Figure 2:**
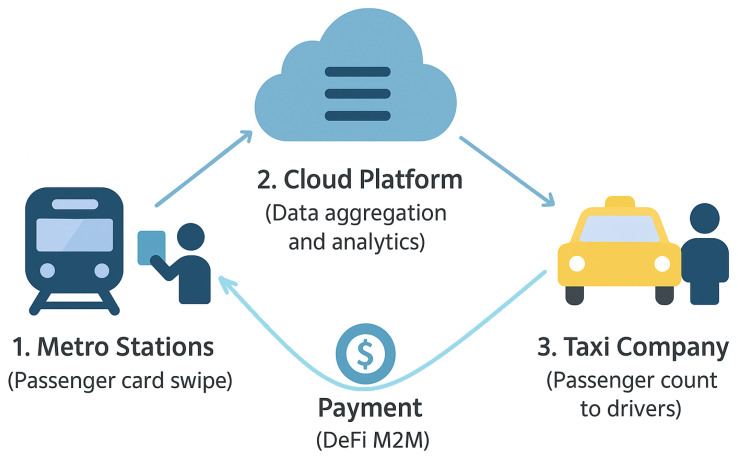
Passenger-flow data sharing system between metro stations, a cloud platform, and a taxi company.

IoT data are increasingly regarded as a new class of digital assets. Trading IoT data through decentralized marketplaces rewards data producers fairly and enables monetization at a granular level [27]. Figure 2 illustrates a real-world example where metro station IoT sensors collect passenger-flow data, which is then monetized through cloud analytics and DeFi-based settlement.

### 3.3. Trustless IoT Service Provisioning

Trustless service provisioning allows IoT services such as data access, actuation, or computing tasks to be delivered without centralized authorities. This relies on blockchain, smart contracts, and decentralized identity frameworks, ensuring verifiable conditions for service execution, transparency, and autonomy.

### 3.4. Supply Chain Finance with Real-Time IoT Tracking

IoT-enabled supply chain finance (SCF) uses real-time tracking to trigger automated payments, tokenized invoices, or micro-loans via DeFi protocols [13,28,29]. Continuous visibility into logistics improves efficiency, reduces fraud, and provides trustless financing.

### 3.5. Insurance Automation Using IoT Data and DeFi Protocols

IoT sensors collect real-time asset data, which can trigger parametric insurance smart contracts for dynamic premium adjustment or claim execution [30,31]. DeFi protocols facilitate decentralized risk pools, reducing reliance on traditional insurers, lowering administrative costs, and providing transparent, trustless operations.

### 3.6. Decentralized Identity (DID) for Devices

DIDs provide IoT devices with self-sovereign identities anchored on blockchain [32]. Devices can authenticate and authorize interactions trustlessly. Verifiable credentials (VCs) allow selective disclosure of device attributes, enabling secure M2M payments, decentralized marketplaces, and trustless service provisioning, while mitigating risks of spoofing and single points of failure.

### 3.7. Energy Trading in Smart Grids Using DeFi

**Figure 3:**
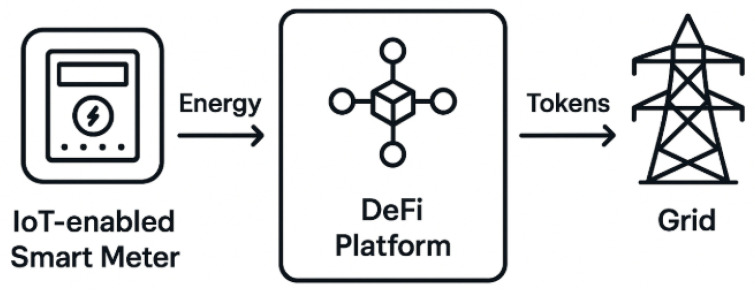
Energy trading process in smart grids using decentralized finance (DeFi).

IoT-enabled smart meters and devices enable decentralized peer-to-peer energy trading. Real-time energy data is verified on-chain, and DeFi smart contracts automate buyer-seller matching and tokenized payments [33,34,35,36]. Figure 3 shows the operational workflow.

### 3.8. Economic Feasibility of IoT–DeFi Micropayments

IoT micropayments are often fractions of a cent, making Layer-1 blockchain fees prohibitively high. Layer-2 solutions, rollups, state channels, or off-chain aggregation reduce per-event costs, enabling high-frequency M2M financial interactions to be economically feasible.

### 3.9. Quantitative Economic Model for IoT–DeFi Micropayments

Let *N* denote the number of IoT events batched. Per-event transaction costs can be modeled as follows:

- Direct L1: $C_{\mathrm{direct}}=C_{L1}$
- L2 batching/rollups: $C_{L2,\mathrm{event}}=\frac{C_{L2}+C_{L1}}{N}$
- Off-chain aggregation: $C_{\mathrm{off},\mathrm{event}}\approx C_{\mathrm{off}}+\frac{C_{L1}}{N}$

This framework evaluates economic feasibility across blockchain architectures [37,38,39,40].

### 3.10. Crowdfunding and Investment for IoT Projects via DeFi

DeFi enables decentralized crowdfunding for IoT projects through token issuance representing equity, usage rights, or governance power. DAOs govern fund allocation and roadmap milestones, reducing intermediaries and empowering the community [41,42].

### 3.11. Use Cases in Smart Cities, Supply Chains, and Autonomous Systems

IoT–DeFi integration supports decentralized micropayments in smart cities (tolls, utilities), supply chains (real-time tracking and automated logistics payments), and autonomous systems (M2M services for drones, vehicles) [42]. Table 3 presents a comparative evaluation of key IoT–DeFi convergence opportunities by analyzing their feasibility, economic considerations, technological readiness levels (TRL), and regulatory or barrier-related constraints. The assessment shows that most use cases are at moderate feasibility with TRL values ranging from 4 to 7, indicating early to mid-stage maturity. While economic benefits such as cost reduction, automation, and fraud mitigation are evident, widespread adoption is constrained by on-chain transaction costs, secure key management requirements, and significant regulatory challenges related to finance, data protection, and cross-border compliance.

## 4. Cross-Layer Security Challenges and Systemic Risk Analysis in IoT–DeFi Integration

Unlike traditional IoT or DeFi systems studied in isolation, the integration of IoT with DeFi introduces systemic risks that arise from the interaction of physical-world data, automated financial logic, and immutable execution environments. Vulnerabilities at the IoT layer such as sensor spoofing, firmware compromise, or device identity manipulation can propagate through oracle mechanisms into smart contracts, triggering irreversible financial actions. In contrast to conventional IoT attacks, which typically impact availability or data integrity, IoT–DeFi failures can directly cause financial loss, market manipulation, or cascading liquidation events.

Similarly, DeFi-native risks such as composability-induced attack propagation, oracle price manipulation, and governance capture are amplified when external IoT data is used as a trigger for financial execution. The absence of robust data provenance and trust metrics transforms noisy or adversarial sensor inputs into systemic economic threats. As a result, IoT–DeFi security must be evaluated not only at the component level, but also from a cross-layer and economic risk perspective, accounting for likelihood, destructiveness, and attack incentives.

### 4.1. Assumptions and Security Boundary Conditions

Any system that relies on data originating from the physical world is subject to an irreducible trust boundary at the sensing interface. Cryptographic mechanisms cannot guarantee the semantic correctness or authenticity of physical-world observations; they can only ensure integrity, provenance, and accountability after data acquisition. Consequently, security in IoT–DeFi systems should not be interpreted as absolute correctness of sensed data, but as the containment of adversarial influence beyond the point of data capture. Similarly, references to secure data transmission denote protection against network-level attacks such as tampering, replay, and unauthorized access, rather than guarantees of truthful sensing. These distinctions define the scope within which decentralization and security claims are made and avoid overextending cryptographic assurances beyond their theoretical limits. Work on hybrid ML-based intrusion detection in cyber-physical manufacturing systems demonstrates how security failures at the operational technology layer can cascade into higher-level financial workflows, underscoring the relevance of layered defenses in IoT-centric DeFi scenarios [43].

### 4.2. Centralization Trade-Offs in IoT–DeFi Architectures

The use of gateways, edge servers, or cloud platforms in IoT–DeFi systems introduces a form of infrastructural centralization that warrants careful consideration. While these components can improve scalability, latency, and feasibility for resource-constrained devices, they may also reintroduce single points of failure, trust concentration, or censorship risk if not properly constrained. Evidence from fog-assisted NB-IoT deployments shows that security-aware resource allocation strategies can reduce latency and improve reliability at intermediate computing layers, reinforcing the importance of edge–fog coordination in IoT–DeFi integration [44,45].

Importantly, infrastructural centralization does not inherently negate decentralization at the protocol level. When gateways act as stateless relays or verifiable computation proxies, and when their outputs are cryptographically authenticated and independently verifiable on-chain, the system can preserve trust-minimized operation despite centralized deployment. Conversely, architectures that rely on trusted gateways without cryptographic accountability represent a genuine compromise to decentralization. As a result, decentralization in IoT–DeFi systems should be evaluated along a spectrum rather than as a binary property, with design choices balancing feasibility and trust minimization.

Table 4 provides a concise overview of various IoT–DeFi integration patterns, highlighting how different roles played by IoT devices align with specific DeFi primitives and the security trade-offs involved. It outlines use cases such as autonomous M2M payments, decentralized data marketplaces, automated IoT service provisioning, supply chain finance, and parametric insurance, emphasizing both their functional benefits and potential vulnerabilities. The table also covers identity management, energy trading, IoT-focused crowdfunding, and smart city coordination, noting key risks such as oracle manipulation, smart contract exploits, privacy leaks, and interoperability challenges. Overall, the table maps how IoT devices interact with DeFi systems while summarizing the security considerations that must be addressed for reliable large-scale deployment.

### 4.3. Smart Contract Vulnerabilities and Exploits

While smart contracts offer automation, transparency, and trustlessness, they are also susceptible to a range of vulnerabilities that can lead to severe financial losses. Common issues include reentrancy attacks, integer overflows/underflows, improper access control, and unchecked external calls. One of the most well-known incidents was the DAO attack in 2016, where an attacker exploited a reentrancy flaw in the Ethereum-based DAO smart contract to siphon approximately $60 million worth of Ether [46,47]. Such exploits have prompted the development of formal verification tools and secure-by-design languages like Vyper and Scilla. However, vulnerabilities continue to surface, as evidenced by numerous DeFi protocol hacks due to flawed logic, improper oracle integration, or liquidity manipulation [47]. Ensuring the reliability of smart contracts, particularly those interacting with IoT systems and DeFi, is critical for the safety and adoption of decentralized technologies.

### 4.4. Consensus Protocol Risks

Consensus protocols are fundamental to maintaining the integrity of blockchain networks, but they introduce specific risks, particularly in IoT–DeFi integrations. Proof-of-work (PoW) mechanisms suffer from high energy consumption and vulnerability to 51% attacks [48]. Proof-of-Stake (PoS) and its variants offer better scalability but are exposed to risks such as long-range attacks, stake centralization, and validator bribery [49]. These vulnerabilities become critical in IoT–DeFi systems, where real-time data from IoT devices triggers financial transactions based on the blockchain state. Delays, forks, or malicious manipulation in consensus can lead to erroneous or unintended outcomes. Additionally, resource-constrained IoT nodes often cannot participate fully in consensus, relying instead on third-party gateways, which may themselves be untrusted [50]. Ensuring consensus robustness and employing trusted or reputation-based intermediaries is essential for maintaining system security and data integrity.

### 4.5. Data Integrity and Oracle Manipulation

In IoT–DeFi systems, data integrity is paramount, as financial smart contracts often rely on external data inputs to trigger actions. Since IoT devices are inherently vulnerable to physical tampering, sensor spoofing, or network-level attacks, malicious or inaccurate data can lead to incorrect or fraudulent smart contract execution. To bridge the gap between off-chain IoT data and on-chain logic, oracles are employed. However, oracles themselves are potential points of failure, susceptible to data manipulation, front-running, or collusion [51]. Notable incidents in DeFi have shown that compromised oracles can lead to multi-million dollar exploits through artificially manipulated prices or event triggers [52]. Ensuring trust in oracles through cryptographic proofs, decentralized oracle networks (e.g., Chainlink), or secure hardware modules is essential to maintaining end-to-end integrity in IoT–DeFi ecosystems [53]. Complementary to these challenges, Nedungadi et al. [54] propose a lightweight hybrid multimodal IDS tailored for edge-enabled IoT environments, demonstrating that resource-aware detection models can significantly mitigate data-layer compromise risks prior to DeFi-triggered execution. Anomaly detection approaches that leverage device-level power-consumption patterns show promise for validating behavioral authenticity of IoT data streams, thereby strengthening the trustworthiness of sensor inputs consumed by DeFi-driven automation [55].

### 4.6. Identity and Access Management

Identity and Access Management (IAM) is a foundational requirement for secure interaction between IoT devices and DeFi protocols. In traditional systems, identity is managed centrally, but in decentralized ecosystems, IAM must be handled through cryptographic primitives and decentralized identifiers (DIDs). Each IoT device or user interacts using public-private key pairs, but managing these at scale introduces complexity, especially for resource-constrained devices [56]. Unauthorized access, identity spoofing, or key compromise can result in fraudulent transactions, data leakage, or denial of service. Emerging solutions leverage blockchain-based identity frameworks, such as self-sovereign identity (SSI), verifiable credentials, and Ethereum Name Service (ENS), to enable trustless, auditable, and fine-grained access control [57,58]. Integrating IAM securely in IoT–DeFi systems is essential to ensure that only authorized devices and users can initiate, approve, or audit financial operations.

### 4.7. Feasibility of ZKP-Based Identity Verification Under IoT Constraints

While zero-knowledge proofs (ZKPs) provide strong privacy and identity guarantees, their direct applicability to highly resource-constrained IoT devices remains limited. The existence of lightweight cryptographic primitives does not imply that full ZKP generation or verification is feasible on ultra-low-power sensors with strict constraints on computation, memory, and energy.

Recent empirical studies demonstrate that ZKP-based authentication is viable primarily in moderately capable embedded devices or through edge-assisted architectures, where computationally intensive proof generation or verification is offloaded to gateways or edge nodes. For example, implementations of zk-SNARK-based authentication on embedded platforms show acceptable performance only when supported by optimized libraries, hardware acceleration, or proxy-based computation.

Consequently, ZKP adoption in IoT–DeFi systems should be viewed as context-dependent rather than universally applicable. In practical deployments, hybrid models—where IoT devices generate lightweight commitments while edge infrastructure performs ZKP operations—offer a more realistic trade-off between privacy, security, and resource efficiency.

For ultra-constrained IoT devices, alternative authentication mechanisms such as lightweight symmetric cryptography, PUF-based identity, or verifiable credentials without full ZKP support may remain preferable. “Future work should empirically benchmark ZKP schemes across representative IoT hardware classes to establish standardized feasibility thresholds.”

#### 4.7.1. Empirical Evidence: ZKPs on Embedded Devices

Although traditional ZKP schemes were considered too heavy for constrained devices, recent advances have demonstrated practical implementations on embedded systems. For example, the work ZPiE shows that state-of-the-art ZKP authentication protocols (e.g., using zk-SNARKs) can be compiled and executed on a variety of embedded systems, yielding acceptable performance even on limited hardware platforms [59]. Similarly, lightweight ZKP-based authentication protocols tailored for embedded IoT devices have been proposed, which reduce computational, storage, and communication overhead compared to conventional cryptographic authentication schemes. These results indicate that ZKP usage is not categorically infeasible for IoT: with careful design, it can be adapted to resource-limited environments.

#### 4.7.2. Trade-Offs Between Proof System Choices

Different ZKP paradigms—non-interactive (e.g., zk-SNARK, Bulletproof) vs. interactive, or classical vs. post-quantum—pose different resource trade-offs. A recent benchmark comparing zk-SNARK, zk-STARK, and Bulletproof in a minimal-hash application shows that zk-SNARK produces the smallest proofs, while zk-STARK yields larger proofs and may incur higher generation/verification cost; this suggests that protocol selection must be carefully matched to IoT constraints and use-case requirements [60]. For real-time or low-latency IoT applications, proof size, generation time, and verification overhead all matter—making lightweight or optimized ZKP protocols more suitable than heavier ones.

#### 4.7.3. Hybrid Architectural Approaches

Given that many IoT endpoints remain severely resource-constrained, fully performing ZKP proof generation or verification on-device may not always be viable. Instead, a hybrid architecture can help: the IoT device performs minimal operations (e.g., generating a commitment, simple symmetric cryptography), while a more capable **edge gateway or proxy** (edge node, IoT gateway, or dedicated verifier) handles heavy ZKP computation or verification. This retains privacy benefits (the identity is not disclosed to the gateway) while amortizing resource costs off-device. Such architectures have been demonstrated in blockchain-based IoT identity frameworks combining ZKP with device-specific credentials or decentralized identifiers (DIDs) and on-chain verification [61].

#### 4.7.4. When ZKP Makes Sense in IoT–DeFi Context

Based on current evidence, ZKP-based identity verification is feasible and appropriate in IoT–DeFi systems under the following conditions:

- The IoT device is either moderately capable (e.g., slightly more powerful embedded hardware) or can offload heavy computation to an edge or gateway node.
- The application demands strong privacy or anonymity—e.g., device identity masking, privacy-preserving credentials, anonymous data contribution—where traditional PKI or identity systems do not suffice.
- The communication and latency budgets allow for the overhead of proof generation and verification (or it is batched/amortized).
- The ZKP protocol is carefully chosen (e.g., optimized SNARKs or lightweight protocols) to match device and network constraints.

Nevertheless, adoption is not without cost. Constraints remain in terms of energy consumption (for battery-powered devices), proof-generation time, memory footprint, and network bandwidth for proof transmission. Moreover, if proof verification occurs off-device (e.g., via a gateway), trust assumptions about the gateway must be carefully considered. Finally, for very low-end or ultra-constrained devices (e.g., tiny sensors with minimal hardware), even lightweight ZKP schemes might remain impractical—in such cases, alternate identity/authentication mechanisms may still be preferable. In conclusion, while IoT devices pose significant resource constraints, recent research demonstrates that ZKP-based identity verification is often feasible when paired with appropriate protocol choices and architectural adaptations. Therefore, recommending ZKPs in IoT–DeFi systems is not unfounded—provided that the limitations are acknowledged and design choices are justified. Future work should empirically evaluate ZKP performance in representative IoT deployments and standardize hybrid architectures suitable for large-scale IoT–DeFi convergence.

### 4.8. Privacy Issues in IoT Transactions

IoT transactions generate vast amounts of sensitive data, including location, biometric, environmental, and behavioral information. When such data is transmitted over decentralized networks or logged on public blockchains as part of DeFi interactions, it raises significant privacy concerns. Unlike traditional systems where access control is centralized, blockchain’s transparency can inadvertently expose transaction metadata, device identifiers, or user behavior. Moreover, resource-constrained IoT devices may lack robust encryption capabilities, increasing the risk of data interception and leakage [62]. Privacy-preserving technologies such as zero-knowledge proofs, homomorphic encryption, and off-chain data handling are being explored to mitigate these risks [63]. However, balancing data transparency for auditability with user privacy remains an ongoing challenge in IoT–DeFi systems.

### 4.9. Financial Exploitation and Attacks

The fusion of IoT and DeFi introduces novel vectors for financial exploitation and cyberattacks. Recent studies on AI-driven detection of obfuscated malware highlight how advanced evasion techniques targeting IoT firmware and middleware could propagate financial exploitation risks when such compromised devices interact with DeFi protocols [64]. Smart contracts that automate payments based on IoT data can be manipulated through compromised sensors, spoofed device identities, or tampered environmental inputs [65]. In DeFi, attackers have exploited vulnerabilities such as reentrancy, flash loan arbitrage, and oracle manipulation to extract millions of dollars, often in seconds. When IoT data feeds are used to trigger financial actions such as insurance payouts, supply chain payments, or energy trades, any attack on data integrity can directly lead to unauthorized fund transfers. These exploits are exacerbated by the irreversible nature of blockchain transactions, the lack of standardized auditing tools for smart contracts, and limited financial protections for users. As IoT devices increasingly serve as financial oracles, ensuring the authenticity and tamper-resistance of data is critical to preventing systemic abuse in IoT–DeFi applications.

### 4.10. Gateway Dependency and Architectural Centralization

Although IoT–DeFi systems strive to achieve trustless and fully decentralized operation, most practical IoT deployments continue to depend on gateway devices such as mobile hubs, edge routers, or proprietary controllers. These gateways often handle protocol translation, authentication, and blockchain communication on behalf of resource-constrained IoT nodes. However, this architectural reliance introduces a decentralization bottleneck—gateways may serve as single points of failure, bottleneck nodes, or privileged trust entities. In addition, a compromised gateway can manipulate sensor readings, inject fabricated data, or filter legitimate device outputs before they are submitted to smart contracts, directly weakening the security assurances of decentralization. Addressing this issue requires exploring designs that reduce or eliminate gateway dependence, including lightweight blockchain light clients on IoT devices, distributed gateway redundancy, and trust-minimized communication protocols.

### 4.11. Conflict Between Blockchain Immutability and the Right to Be Forgotten

One emerging challenge in IoT–DeFi integration is the tension between *blockchain immutability* and legal frameworks such as the “Right to be Forgotten” (RTBF) is defined in data protection regulations (e.g., GDPR). IoT devices continuously generate sensitive personal data, including location traces, biometric values, and behavioral patterns. When such data, metadata, or hashed identifiers are anchored to a blockchain for integrity or auditability, they become effectively irreversible due to the append-only and tamper-resistant nature of distributed ledgers. This creates a regulatory conflict: while IoT users may request deletion of their personal data under RTBF, blockchain systems are designed such that stored records cannot be modified or erased. Even if only hashed or pseudonymized IoT data is stored, certain jurisdictions still consider it as personal data if re-identification is possible.

Existing approaches attempt to mitigate this tension by storing privacy-sensitive IoT data *off-chain* and placing only cryptographic commitments or revocable references on-chain. Techniques such as chameleon hashes, redactable blockchains, and proxy re-encryption have been proposed to introduce conditional mutability, but these mechanisms introduce new trust assumptions or computational overheads that may not be suitable for constrained IoT environments. Therefore, achieving compliance with RTBF while maintaining the decentralized security guarantees required by DeFi remains an open challenge in IoT–DeFi system design.

### 4.12. Limited Hardware Interfaces

IoT devices are typically constrained by limited hardware resources such as low processing power, memory, battery capacity, and communication bandwidth. These limitations present significant challenges when integrating such devices with blockchain-based DeFi systems, which often require cryptographic operations, smart contract interaction, and data validati. Most IoT nodes cannot run full blockchain clients due to storage and computational constraints, leading to reliance on third-party gateways or lightweight protocols. This dependency can undermine decentralization and expose the system to trust assumptions or single points of failure. Furthermore, secure key storage and transaction signing on constrained hardware remain unresolved issues, increasing the risk of unauthorized access and transaction manipulation [66]. Designing energy-efficient, secure, and scalable interfaces is essential to enable robust and practical IoT-DeFi deployments.

### 4.13. A Risk Assessment of Security Challenges

A systematic risk assessment is essential for understanding how IoT–DeFi integration exposes systems to multi-layered vulnerabilities. Because risks arise simultaneously from IoT hardware constraints, blockchain execution environments, and adversarial financial incentives, their evaluation must consider three dimensions, as follows: likelihood, destructiveness, and attacker cost.

#### 4.13.1. Likelihood of Attacks

In the IoT domain, attacks exploiting weak device authentication, unsecured wireless channels, and firmware vulnerabilities are classified as high-likelihood events because IoT nodes often lack tamper-resistant hardware and operate with outdated security controls [67]. On the DeFi side, smart contract bugs (e.g., reentrancy, integer overflows) and oracle manipulation are also high-likelihood due to numerous documented incidents across major protocols. When IoT devices feed real-time data to DeFi protocols, these vulnerability classes compound, making cross-layer attacks such as oracle spoofing through compromised sensors or manipulated device identities significantly more probable.

#### 4.13.2. Destructiveness of Risks

The destructiveness of IoT–DeFi security breaches is magnified by the financial finality of blockchain transactions. Attacks against IoT data or device identities can trigger unauthorized fund transfers, liquidation cascades in lending platforms, or fraudulent insurance payouts. Oracle-level manipulation is considered high impact because adversaries can directly alter the economic behavior of DeFi protocols, generating losses ranging from millions to billions. Compared to traditional IoT breaches which typically compromise privacy or availability the integration with DeFi introduces direct monetary consequences and systemic risks to liquidity pools and automated market makers (AMMs).

#### 4.13.3. Cost of Attack Implementation

The cost of executing IoT-layer attacks is generally low because many IoT devices lack secure boot, hardware enclaves, or strong cryptographic modules. Physical access to devices enables low-cost firmware extraction or sensor spoofing [68]. DeFi-related attacks vary—exploiting poorly audited or immature smart contracts may require moderate technical expertise but negligible capital expenditure. However, economically driven attacks such as oracle price manipulation or flash-loan-based exploits may demand substantial liquidity to influence on-chain prices, though flash loans significantly reduce upfront cost barriers. In the IoT–DeFi convergence, adversaries can combine low-cost IoT compromises with high-leverage DeFi mechanisms, creating asymmetric attack profiles where the cost to the attacker is minimal but the financial impact is severe.

#### 4.13.4. Overall Risk Posture

Evaluating the combined threat model suggests that IoT–DeFi systems exhibit a high-likelihood, high-impact, low-to-moderate-cost risk profile. This combination is dangerous: attackers can compromise inexpensive IoT endpoints to influence high-value DeFi operations. Effective mitigation, therefore, requires integrated defenses spanning device hardening, oracle security, identity management, and smart contract resilience. Without holistic approaches, attackers may exploit the weakest component—typically an IoT endpoint—to cause disproportionate financial and operational damage.

## 5. Existing Mitigation Techniques

While numerous security mechanisms have been proposed for IoT and DeFi independently, their effectiveness in integrated IoT–DeFi environments varies significantly depending on device capability, trust assumptions, and attack surface. Rather than enumerating countermeasures, we evaluate mitigation strategies based on their ability to (i) prevent attack propagation, (ii) reduce economic impact, and (iii) preserve decentralization under resource constraints.

### 5.1. Lightweight Cryptographic Techniques

Lightweight cryptographic techniques are essential for securing resource-constrained IoT devices interacting with DeFi protocols. Traditional algorithms such as RSA-2048 or standard ECC introduce high computational and energy overheads that are infeasible for embedded devices. Lightweight alternatives, including PRESENT, SPECK, SIMON, Trivium, and ASCON, significantly reduce memory footprint, energy consumption, and execution latency [69].

*Lightweight vs. heavyweight cryptography mismatch:* IoT devices typically employ lightweight cryptographic primitives to accommodate strict constraints on computation, memory, and energy consumption. In contrast, DeFi protocols commonly rely on computationally intensive security mechanisms such as ECDSA verification, multi-signature schemes, and zero-knowledge proof systems. This asymmetry can create interoperability challenges: resource-limited IoT nodes may struggle to perform the cryptographic operations required to authenticate, sign, or validate complex DeFi transactions, potentially introducing latency or offloading burdens to gateways or edge nodes. Consequently, additional cryptographic translation layers, hardware accelerators, or off-chain verification mechanisms may be needed to mitigate these bottlenecks and ensure efficient cross-domain interaction. Integrity-preserving MAC frameworks such as EP-CuMAC illustrate that carefully engineered lightweight cryptographic protection can secure constrained NB-IoT communications without incurring prohibitive performance overheads in DeFi-linked device transactions.

*Performance and trade-offs:* For example, PRESENT-80 requires only 1570 GE in hardware and consumes less than 5 μW [70], whereas Trivium achieves 2–3 Mbps throughput with fewer than 3000 GE. Lightweight ECC variants can reduce signature generation time by over 60% compared to standard ECC [71]. These optimizations make frequent M2M micro-transactions and smart contract interactions feasible in IoT–DeFi ecosystems. However, they often provide lower security margins compared to traditional cryptography, requiring careful parameter selection.

*Integration and deployment:* Lightweight cryptography supports secure device identity, transaction signing, and message integrity in IoT–DeFi systems [72]. Deployment challenges include secure key management [73], compatibility with blockchain signature schemes [74], and maintaining performance under high-frequency event streams [75]. Hardware-assisted modules (e.g., TPM, HSM) [76] and PUF-based authentication [77] can further strengthen security without exceeding energy budgets. Table 5 compares the performance of representative lightweight cryptographic algorithms suitable for IoT–DeFi devices in terms of hardware memory footprint, throughput or latency, and energy consumption per operation. The results indicate that symmetric primitives such as PRESENT-80 and Trivium offer low gate-equivalent (GE) requirements and minimal power consumption, making them well suited for resource-constrained IoT nodes.

### 5.2. Secure Smart Contract Development

Smart contracts are central to DeFi operations but are vulnerable to logic errors and attacks. Secure development practices include modular code design, least-privilege access control, and adoption of security-focused languages such as Vyper or Move [78,79].

Formal Verification and Auditing: Tools like Certora, Feist2019, and MythX allow static and symbolic analysis to detect vulnerabilities prior to deployment [80,81,82]. Regular audits, combined with defensive coding patterns, reduce attack surfaces and improve resilience.

Integration in IoT–DeFi: IoT devices interacting with smart contracts require low-latency, secure transactions. Smart contracts must handle oracle delays, micropayment batching, and event-triggered automation without introducing execution bottlenecks. Multi-signature wallets, circuit breakers, and upgradeable proxies enhance trust and safety in distributed IoT financial networks.

### 5.3. Blockchain-Oriented Identity Management

Decentralized identity (DID) frameworks, Self-Sovereign Identity (SSI), and verifiable credentials (VCs) mitigate centralized trust risks [83,84]. These approaches allow IoT devices to authenticate and authorize transactions in a privacy-preserving manner.

Performance and limitations: Lightweight cryptography combined with DIDs allows devices to maintain secure identities with minimal overhead. Challenges include key management, interoperability across blockchains, and compliance with privacy regulations (e.g., GDPR).

Integration in IoT–DeFi: Devices can prove credentials to smart contracts for access to DeFi services without revealing unnecessary data. This enables autonomous M2M payments, trustless data marketplaces, and decentralized service provisioning while reducing single points of failure.

### 5.4. Oracle Security Mechanisms

Oracles provide external data to DeFi smart contracts but introduce risks like manipulation, downtime, or Sybil attacks. Mitigation approaches include decentralized oracle networks (DONs), trusted execution environments (TEEs), multi-party computation (MPC), and zero-knowledge proofs (ZKPs) [85].

Performance and trade-offs: Decentralized oracles improve reliability but increase latency. TEEs and ZKPs enhance data integrity but consume additional computation and energy. Reputation and scoring systems can further discourage malicious behavior.

### 5.5. Protocol-Level Risk Mitigation in DeFi

DeFi protocols are exposed to liquidity risks, governance attacks, and smart contract bugs [86]. Mitigation strategies include:

- Formal verification and auditing of contracts.
- Oracle redundancy and time-weighted average pricing (TWAP).
- Automated circuit breakers, pause functions, and liquidity reserves.
- Secure governance with quorum requirements, time-locks, and gradual parameter changes.

Integration with IoT–DeFi: These strategies ensure that automated IoT-triggered financial transactions remain reliable, even under volatile market conditions or attempted exploits.

### 5.6. Limitations in Resource-Constrained Environments

IoT devices face significant constraints on processing, memory, and energy, limiting direct blockchain interaction [87,88]. Lightweight cryptography, off-chain computation, and edge-assisted consensus models are essential to balance trust and efficiency. Trade-offs exist between decentralization, performance, and energy consumption, requiring adaptive architectures tailored to specific IoT–DeFi scenarios.

## 6. Research Gaps and Open Challenges

### 6.1. Need for Adaptive Trust and Reputation Models

While trust and reputation mechanisms exist for decentralized systems, current models are largely static and fail to capture the dynamic, context-dependent behavior of participants in large-scale IoT–DeFi ecosystems [89]. Key research gaps include:

- Dynamic context modeling: Existing models rarely account for temporal behavior patterns, environmental factors, or cross-layer interactions among devices and smart contracts.
- Attack resilience: Many schemes are vulnerable to Sybil attacks, collusion, or coordinated misbehavior. Metrics for quantifying robustness are underdeveloped.
- Integration with governance: Current approaches do not link adaptive trust scores with protocol-level decision-making or risk mitigation in real-time.

Future work should focus on developing machine learning-based adaptive trust systems with quantifiable metrics (e.g., false-positive/negative rates, update latency), simulation frameworks for adversarial scenarios, and protocols that integrate trust assessments into decentralized governance and resource allocation.

### 6.2. Interoperability Among Heterogeneous IoT–DeFi Systems

Interoperability remains a critical barrier to the practical integration of IoT devices with DeFi platforms [90,91]. Identified research gaps include the following:

- Semantic and protocol-level interoperability: While some cross-chain bridges exist, mechanisms to unify heterogeneous IoT data formats, event triggers, and smart contract interfaces are lacking.
- Performance metrics: There is no standardized framework for measuring integration latency, transaction throughput, or reliability in cross-domain workflows.
- Middleware and API design: Lightweight, modular middleware capable of translating between IoT protocols and blockchain platforms is underexplored.

Research directions should prioritize formal models for semantic interoperability, benchmark suites for end-to-end integration performance, and adaptive middleware supporting heterogeneous devices and blockchain protocols.

### 6.3. Resilient and Efficient Identity Frameworks

Decentralized identity (DID) frameworks are promising but insufficiently optimized for resource-constrained IoT devices [92]. Open challenges include:

- Fault tolerance: Efficient recovery, key rotation, and revocation mechanisms are underdeveloped for devices prone to disconnection or compromise.
- Resource-aware cryptography: Lightweight cryptographic operations must balance security with low memory and energy usage.
- Standardization and Interoperability: Cross-chain and cross-domain identity verification protocols lack unified standards.

Research should investigate edge-assisted DID management, quantifiable performance-security trade-offs, and composable frameworks combining verifiable credentials, zero-knowledge proofs, and biometric verification. Table 6 summarizes the key research gaps and open challenges in IoT–DeFi systems, highlighting each gap’s description and assigned priority, with high-priority areas including adaptive trust models, interoperability, privacy-preserving protocols, and robust oracle design [93].

### 6.4. Privacy-Preserving Protocols

Ensuring privacy in IoT–DeFi systems is challenging due to the transparency requirements of blockchains [94]. Key gaps include:

- Efficiency vs. privacy: Current privacy-preserving protocols often incur high computation and latency costs, making them impractical for real-time IoT applications.
- Selective disclosure: Mechanisms for attribute-based verification without revealing unnecessary data are under-optimized.
- Composability: Integration of privacy protocols across multiple blockchain layers and IoT endpoints is limited.

Future research should explore lightweight zk-SNARK/zk-STARK variants, benchmarking frameworks for IoT-friendly privacy protocols, and hybrid approaches combining on-chain and off-chain privacy mechanisms.

### 6.5. Robust Oracle Design for IoT Contexts

Oracles remain a critical vulnerability point when bridging IoT data to DeFi smart contracts [95]. Research gaps include:

- Resource-constrained deployment: Existing DONs and TEEs are often too heavy for low-power IoT devices.
- Latency-sensitive data: Few protocols guarantee the timely delivery of sensor data for real-time automation.
- Security metrics: Lack of standardized measures for data integrity, fault tolerance, and resistance to coordinated attacks.

Research should prioritize edge-assisted oracle designs, adaptive aggregation and redundancy methods, and formal models for evaluating oracle reliability in dynamic IoT contexts.

### 6.6. Secure Integration Testing Frameworks

Current testing frameworks are insufficient for evaluating end-to-end security and interoperability in IoT–DeFi systems [96]. Open challenges include:

- Cross-layer threat simulation: Few tools can emulate adversarial conditions spanning smart contracts, oracles, and IoT devices.
- Benchmarking and metrics: Standardized security and performance benchmarks for integrated deployments are missing.
- Automated verification: Integration of fuzzing, symbolic execution, and formal verification across heterogeneous components is limited.

Future work should focus on modular, automated testing platforms, quantitative risk assessment methods, and frameworks enabling pre-deployment evaluation of IoT–DeFi applications under adversarial conditions.

## 7. Future Research Directions

Future research should focus on enhancing the scalability, interoperability, and privacy of integrated IoT–DeFi systems. Key areas include the development of lightweight consensus algorithms suitable for edge environments, adaptive trust and reputation mechanisms, and secure cross-chain communication protocols. Sustainability-orientated analyses of cybersecurity using ML-based topic modeling further emphasize the need to align IoT–DeFi security architectures with broader energy-efficiency and socio-technical governance considerations [97]. Further exploration into privacy-preserving identity frameworks, robust oracle designs, and resilient governance models is also essential. Emerging technologies such as federated learning, zero-knowledge proofs, and AI-driven threat detection can be integrated with blockchain. Their combination creates promising avenues for designing decentralized ecosystems that are more secure, autonomous, and intelligent.

### 7.1. Formal Methods for IoT-Integrated Smart Contracts

Formal methods offer mathematically rigorous techniques to specify, verify, and validate the correctness of smart contracts, especially in IoT-integrated environments where security and reliability are critical. As IoT devices autonomously trigger smart contract executions, even minor flaws in contract logic can lead to irreversible actions or financial loss [98]. Techniques such as model checking, theorem proving, and symbolic execution can be applied to formally verify contract properties, including safety, liveness, and access control. Tools like KEVM, Isabelle/HOL, Coq, and VeriSolid support the development of provably correct smart contracts. Integrating these tools with IoT middleware enhances end-to-end trust, ensuring that data from constrained devices triggers only formally validated logic on the blockchain. Future work should focus on scalable verification for modular contracts and context-aware IoT inputs to ensure correctness under dynamic operational conditions.

### 7.2. Edge-AI and Blockchain Synergies

The convergence of edge artificial intelligence (edge-AI) and blockchain technologies presents a powerful paradigm for building intelligent, secure, and autonomous cyber-physical systems. Edge-AI enables real-time data processing and decision-making at the network edge, reducing latency, conserving bandwidth, and enhancing privacy by avoiding raw data transmission [99]. When integrated with blockchain, it provides tamper-proof audit trails, decentralized trust, and secure coordination among distributed edge nodes [100]. This synergy is particularly valuable in IoT contexts, where local AI inference (e.g., anomaly detection or predictive maintenance) can trigger blockchain-verified actions such as smart contract execution or logging of critical events [101]. Furthermore, blockchain enhances federated learning by securing model updates and ensuring integrity in collaborative training across edge devices. Despite the promise, challenges remain in achieving resource-efficient consensus, data provenance verification, and seamless integration of heterogeneous platforms, making this a rich area for ongoing research.

### 7.3. Decentralized Identity with IoT Constraints

Decentralized identity (DID) systems offer a self-sovereign model for managing digital identities without reliance on centralized authorities. However, integrating DID frameworks into IoT environments introduces unique challenges due to device-level constraints in computation, memory, energy, and connectivity. Standard DID operations, such as cryptographic key generation, signature verification, and credential management, are often too resource-intensive for low-power edge devices. Furthermore, ensuring secure storage and recovery of private keys in physically exposed IoT nodes remains an unsolved problem. Lightweight identity protocols, hierarchical key structures, and offloading operations to trusted edge gateways are promising approaches to mitigate these constraints [102]. Additionally, incorporating privacy-preserving features such as zero-knowledge proofs and selective disclosure enhances data minimization and compliance with privacy regulations. Future research must balance decentralization, usability, and device-level efficiency to realize scalable and secure identity management for IoT-enabled ecosystems.

The integration of decentralized identity (DID) into Internet of Things (IoT) ecosystems is rapidly emerging as a cornerstone for secure and autonomous device-to-device interactions. However, the inherently constrained nature of IoT environments, characterized by limited processing power, restricted memory, energy constraints, and intermittent connectivity, poses significant challenges for applying conventional identity management systems. DID frameworks, which were originally designed for resource-rich settings, cannot be directly deployed on lightweight IoT nodes without targeted adaptation. Recent advances demonstrate that lightweight distributed ledger technologies, such as IOTA’s Tangle with its Streams and Stronghold components, enable even resource-limited platforms like Raspberry Pi to generate and manage decentralized identifiers, thereby offering a viable pathway for scalable IoT adoption. At the same time, self-sovereign identity (SSI) paradigms are evolving to accommodate IoT-specific requirements, including credential validity, lifecycle governance, and interoperability across heterogeneous networks.

Privacy considerations remain equally pressing—while blockchain-based identity systems enhance auditability and trust, the inherent transparency of distributed ledgers risks exposing sensitive device metadata [103,104]. Approaches such as Enhanced Privacy ID (EPID) and selective disclosure techniques are therefore essential for enabling verifiable, privacy-preserving authentication and onboarding of devices. Taken together, these developments indicate that the next generation of IoT identity frameworks will need to embrace lightweight cryptographic methods, hybrid on-chain/off-chain models, and privacy-by-design principles in order to achieve scalability, efficiency, and trustworthiness within decentralized IoT–DeFi ecosystems.

### 7.4. Post-Quantum Security Considerations

The advent of quantum computing poses a significant threat to current cryptographic primitives that underpin blockchain and decentralized systems. Algorithms such as RSA, ECDSA, and ECC—widely used in smart contracts, digital signatures, and identity frameworks—can potentially be broken by Shor’s algorithm on a sufficiently powerful quantum computer [105]. This vulnerability is particularly critical for long-lived IoT devices and immutable blockchain records that require forward secrecy and long-term data integrity. Post-quantum cryptography (PQC) aims to develop quantum-resistant algorithms, such as lattice-based, hash-based, and multivariate signature schemes [106]. However, the integration of PQC into resource-constrained environments like IoT remains challenging due to the increased key sizes, computational demands, and lack of standardized lightweight implementations. Future research must explore hybrid cryptographic approaches, efficient quantum-safe identity mechanisms, and scalable transition strategies to prepare decentralized infrastructures for the post-quantum era.

### 7.5. Scalable Layer-2 and Cross-Chain Protocols

As blockchain networks grow in adoption and complexity, scalability and interoperability become critical bottlenecks, especially in applications involving high-frequency microtransactions and heterogeneous IoT ecosystems. Layer-2 solutions—such as payment channels, sidechains, rollups, and state channels—offload transaction processing from the base layer, thereby improving throughput, reducing latency, and minimizing transaction costs [107]. Techniques like optimistic and zero-knowledge rollups (zk-rollups) offer scalable smart contract execution while preserving security guarantees by periodically settling on the main chain [108]. In parallel, cross-chain protocols and bridges enable asset and data transfers across disparate blockchain networks, supporting broader interoperability and liquidity aggregation. However, challenges such as trust assumptions, finality mismatches, and bridge vulnerabilities remain significant barriers. Future research should focus on decentralized interoperability standards, verifiable cross-chain communication, and formal verification of bridge logic to ensure secure and scalable blockchain interconnectivity.

### 7.6. DeFi and Federated Learning for Privacy

The convergence of *decentralized finance (DeFi)* and *federated learning (FL)* presents a promising pathway for enhancing privacy-preserving intelligence in decentralized financial systems. DeFi leverages blockchain technologies to enable transparent, permissionless, and trustless financial transactions without intermediaries [109]. On the other hand, FL enables multiple clients to collaboratively train machine learning models across distributed data sources without exposing raw data, thus preserving user privacy [110]. The integration of FL into DeFi applications supports secure and private analytics, such as credit scoring or fraud detection, while maintaining data locality. For instance, users’ devices can participate in model training to predict loan default risk or optimize asset allocation strategies, all without sharing personal financial records with centralized servers [111]. Blockchain infrastructure ensures that model updates and training contributions are verifiable, traceable, and incentivized via smart contracts. Privacy can be further enhanced by incorporating techniques such as *secure multiparty computation (SMPC)*, *differential privacy*, and *zero-knowledge proofs (ZKPs)* to protect intermediate model gradients and updates during training [112]. These tools help maintain the confidentiality of training data while enabling collective learning over distributed sources.

This approach is especially valuable in the context of IoT-enabled financial systems, where billions of edge devices generate sensitive data. Use cases include privacy-preserving decentralized insurance, autonomous machine-to-machine (M2M) payments, and predictive modeling for energy or resource credits. Together, FL and DeFi facilitate a more secure, decentralized, and privacy-aware digital economy. Although this paper provides a comprehensive survey of IoT–DeFi integration, it has several limitations. The scope of coverage is restricted to selected use cases such as smart contracts, oracles, M2M payments, and energy efficiency, leaving other domains like healthcare and industrial finance only briefly addressed. Data availability is limited, as most evidence relies on secondary sources and small-scale prototypes rather than large-scale deployments. Moreover, the rapid technological evolution of both IoT and DeFi may render parts of this study outdated as new standards and architectures emerge. The discussion on security and privacy remains largely conceptual without empirical validation, and regulatory as well as user adoption challenges are acknowledged but not analyzed in depth, which may influence the practical applicability of IoT–DeFi systems.

## 8. Conclusions

The convergence of the Internet of Things (IoT) and decentralized finance (DeFi) represents a transformative shift in the way digital services, economic interactions, and autonomous systems are designed and deployed. This survey has systematically explored the underlying technologies, integration opportunities, security challenges, and future research directions that define this emerging interdisciplinary domain. We began by establishing the foundations of IoT and DeFi, highlighting their complementary characteristics: IoT’s ability to collect and act on real-world data and DeFi’s capacity to autonomously manage value and enforce logic through blockchain-based smart contracts. Through this lens, we identified a wide array of application opportunities, such as autonomous machine-to-machine (M2M) payments, decentralized data marketplaces, and real-time supply chain finance, all of which can reshape industries from logistics to energy to smart cities.

However, the integration of these two domains introduces a range of security, privacy, and scalability challenges. Issues such as smart contract vulnerabilities, oracle manipulation, identity management, and the constraints of IoT hardware create complex attack surfaces that demand robust mitigation techniques. We reviewed existing countermeasures, including lightweight cryptography, secure smart contract practices, and blockchain-enhanced identity frameworks, while acknowledging the limitations these solutions face in highly constrained or heterogeneous environments.

In identifying current research gaps, we underscored the need for interoperable, adaptive, and privacy-preserving frameworks that can operate reliably across decentralized and resource-constrained infrastructures. Challenges such as the lack of resilient identity systems, the complexity of oracle design in dynamic IoT contexts, and the absence of standardized integration testing frameworks remain significant hurdles. We proposed a range of future research directions to advance this field. These include the adoption of formal verification methods for smart contracts, the synergy between edge-AI and blockchain for localized intelligence, the development of decentralized identity mechanisms tailored to IoT constraints, and the application of post-quantum cryptographic primitives. Notably, we highlighted the promising intersection of DeFi and federated learning as a privacy-preserving solution for data-driven financial intelligence.

In summary, this survey provides a foundational map of the IoT–DeFi landscape, serving as a resource for researchers, developers, and policymakers. By addressing current limitations and exploring innovative pathways, the integration of IoT and DeFi holds the potential to redefine trust, automation, and financial interaction in the digital age.

## Figures and Tables

**Table 1:** Proposed IoT–DeFi taxonomy dimensions.

Dimension	Description
Architecture	Classifies system architecture as centralized, hybrid, or fully decentralized, indicating the level of trust assumptions and control.
Data flow models	Describe how IoT data is collected, aggregated, and transmitted to DeFi components (e.g., direct on-chain, off-chain, or via middleware).
Incentive mechanisms	Identify tokenomics, micropayment schemes, staking, or reward systems used to motivate IoT device participation and data sharing.
Consensus protocols	List the blockchain or distributed ledger consensus mechanisms employed (PoW, PoS, DAG-based, lightweight, or hybrid consensus).
Security features	Captures cryptographic techniques, authentication schemes, oracle security, and privacy-preserving mechanisms integrated into the system.
Supported IoT Verticals	Highlight the application domains such as smart cities, logistics, energy management, autonomous vehicles, or healthcare IoT.

**Table 3:** Evaluation of IoT–DeFi convergence opportunities considering feasibility, economic constraints, technological readiness, and regulatory barriers.

Opportunity	Feasibility	Economic Considerations	TRL	Regulatory/Barrier Considerations
Autonomous M2M payments	Moderate; depends on secure key management	Micropayment < transaction cost; Layer-2/off-chain needed	5–6	Financial regulations, cross-border issues
Decentralized data marketplaces	Moderate; relies on aggregation and secure oracles	Data monetization possible, but on-chain cost high	5–6	GDPR, CCPA, IP, interoperability standards
Trustless IoT service provisioning	Moderate; device heterogeneity	Service pricing must cover overhead	5	Sector-specific compliance (healthcare, industrial)
Supply chain finance with IoT tracking	Moderate; integration challenging	Reduced fraud; IoT infrastructure required	6–7	Trade finance, cross-border legal frameworks
Insurance automation via IoT & DeFi	Experimental; sensor/oracle-dependent	Lower cost; potential mispricing risk	4–5	Insurance regulations, liability allocation
Decentralized identity (DID) for Devices	Moderate; secure key management required	Minimal cost; adds security overhead	5	Identity laws, cross-border interoperability
Energy Trading in smart grids	Moderate; needs reliable metering	Micropayment feasibility depends on Layer-2	6	Energy regulations, grid standards, cybersecurity
IoT–DeFi micropayments	Feasible with batching/off-chain aggregation	L1 costs high; Layer-2/off-chain reduces cost	5–6	Network congestion, fee volatility
Crowdfunding IoT projects via DeFi	Feasible in regulated jurisdictions	Tokenized equity/usage rights add volatility	5	Securities law, investor protection, DAO risks
Smart cities/autonomous systems	Moderate; pilot projects feasible	Costs depend on sensor network scale	5–7	Cybersecurity, privacy, municipal regulations

**Table 4:** Condensed mapping of IoT–DeFi integration approaches and their associated security considerations.

Integration Pattern, Device Role, DeFi Primitive	Trade-Offs/Security Notes
M2M Payments—IoT devices as autonomous agents; stablecoins, payment channels	Low latency, lightweight consensus needed; risk of double-spending and micropayment channel attacks.
Decentralized data marketplaces—IoT devices as data providers; tokenization, DEX	Enables data monetization; vulnerable to integrity issues and oracle manipulation.
IoT service provisioning—IoT devices as service executors; Smart contracts, DAOs	Automates SLAs; exposed to smart contract exploits and denial-of-service.
Supply chain finance—IoT devices as asset trackers; Asset-backed tokens, Stablecoins	Increases transparency and trust; requires a secure identity and resilient oracles.
Insurance automation—IoT devices as data oracles; Parametric insurance contracts	Reduces claim disputes; risks of data spoofing and privacy leaks.
Decentralized identity (DID)—IoT devices as identity holders; DID standards, ZK-proofs	Strengthens authentication; constrained by device resources for advanced crypto.
Energy trading—IoT devices as prosumers; AMM-based DEX, Stablecoins	Enables peer-to-peer settlement; subject to volatility and front-running attacks.
Crowdfunding for IoT projects—IoT devices as campaign verifiers; Token issuance, DeFi lending	Democratizes funding; vulnerable to Sybil attacks and fraudulent campaigns.
Smart cities/autonomous systems—IoT devices as coordinators; DAOs, cross-chain bridges	Large-scale coordination is possible; interoperability and scalability issues remain.

**Table 5:** Comparative performance of representative lightweight cryptographic algorithms for IoT–DeFi devices.

Algorithm	Memory (GE)	Throughput/Latency	Energy per Operation
PRESENT-80	1570 GE	1–2 Mbps	<5 μW
Trivium	3000 GE	2–3 Mbps	4–5 μW
ECC-163	–	<15 ms/signature	1.2 mJ
ASCON-AEAD	–	<1 ms/message	1.5 μJ

**Table 6:** Research gaps and open challenges in IoT–DeFi systems with priority ranking.

Research Gap	Description	Priority
Adaptive trust and reputation models	Need for context-aware, dynamic trust evaluation to detect malicious behaviors, collusion, and Sybil attacks in open IoT–DeFi networks.	High
Interoperability among heterogeneous systems	Integration of diverse IoT devices and DeFi protocols with incompatible data formats, communication standards, and consensus mechanisms. Requires standard APIs, bridges, and semantic frameworks.	High
Resilient and efficient identity frameworks	Decentralized identity systems that are fault-tolerant, lightweight, privacy-preserving, and compatible with resource-constrained IoT devices.	Medium
Privacy-preserving protocols	Use of zero-knowledge proofs, homomorphic encryption, MPC, and selective disclosure to protect sensitive data in IoT–DeFi ecosystems.	High
Robust oracle design for IoT contexts	Ensure authentic, timely, fault-tolerant, and low-power data delivery from IoT devices into smart contracts.	High
Secure integration testing frameworks	Comprehensive testing, including adversarial simulation, cross-layer threats, formal verification, and automated coverage tracking.	Medium

## Data Availability

The original contributions presented in this study are included in the article. Further inquiries can be directed to the corresponding author.
